# Achieving Brazil's Deforestation Target Will Reduce Fire and Deliver Air Quality and Public Health Benefits

**DOI:** 10.1029/2022EF003048

**Published:** 2022-12-05

**Authors:** Edward W. Butt, Luke Conibear, Callum Smith, Jessica C. A. Baker, Richard Rigby, Christoph Knote, Dominick V. Spracklen

**Affiliations:** ^1^ School of Earth and Environment University of Leeds Leeds UK; ^2^ Model‐based Environmental Exposure Science Faculty of Medicine University of Augsburg Augsburg Germany

## Abstract

Climate, deforestation, and forest fires are closely coupled in the Amazon, but models of fire that include these interactions are lacking. We trained machine learning models on temperature, rainfall, deforestation, land‐use, and fire data to show that spatial and temporal patterns of fire in the Amazon are strongly modified by deforestation. We find that fire count across the Brazilian Amazon increases by 0.44 percentage points for each percentage point increase in deforestation rate. We used the model to predict that the increased deforestation rate in the Brazilian Amazon from 2013 to 2020 caused a 42% increase in fire counts in 2020. We predict that if Brazil had achieved the deforestation target under the National Policy on Climate Change, there would have been 32% fewer fire counts across the Brazilian Amazon in 2020. Using a regional chemistry‐climate model and exposure‐response associations, we estimate that the improved air quality due to reduced smoke emission under this scenario would have resulted in 2,300 fewer deaths due to reduced exposure to fine particulate matter. Our analysis demonstrates the air quality and public health benefits that would accrue from reducing deforestation in the Brazilian Amazon.

## Key Points

- Using a machine learning approach, we show the spatiotemporal pattern of fire counts in the Amazon is strongly modified by deforestation
- We show increases in deforestation in the Brazilian Amazon between 2013 and 2020 were responsible for a 42% increase in fire counts in 2020
- We estimate 32% fewer fire counts and improved air quality health outcomes in 2020 if Brazil had achieved their Amazon deforestation target

## 1 Introduction

Fire in the Amazon is influenced by both human activity and climate (Aragão et al., 2018; Butt et al., 2021; Cochrane and Barber, 2009; Libonati et al., 2021; Silveira et al., 2020) but the strength of these relationships are still uncertain (Feldpausch et al., 2022). Droughts are strongly correlated with greater fire counts across the Amazon (Aragão et al., 2007, 2018), increasing fire risk through reduced ground water and surface humidity (Ray et al., 2005). People use fires across the Amazon to create and maintain agricultural land (Cochrane, 2003; Morton et al., 2008). Forests are cut and the vegetation is left to dry before fires are lit to clear vegetation. The number of fires is therefore greater in years with more deforestation (Arãgao et al., 2008; Aragão et al., 2018; Chen et al., 2013; Reddington et al., 2015).The rate of deforestation in the Amazon has varied markedly over the past few decades (Hansen et al., 2013; Junior et al., 2020) but little is known about how this has impacted the occurrence and frequency of fire in recent years. Fires emit substantial quantities of carbon dioxide (Withey et al., 2018) and other air pollutants contributing to climate change, degrading air quality and damaging human health (Reddington et al., 2015). The complex interplay between climate, vegetation, and people make it challenging to establish clear links between deforestation and fire. A much clearer understanding of this link is therefore needed to inform sustainable management of the Amazon.

Despite the close links between deforestation, climate and fire, there are few spatially explicit models of fire for the Amazon region that capture both climatic and anthropogenic causes of fire (Silvestrini et al., 2011). Statistical models have been developed that predict fire activity based on sea surface temperature anomalies in the tropical Atlantic and Pacific (Chen et al., 2013; Fernandes et al., 2011; Lima et al., 2018). However, such models based solely on climate miss the important role of people in altering fire across the Amazon. Other models have also been developed to predict burned area using information on socio‐economic features (e.g., population density or gross domestic product) and land cover (Bistinas et al., 2014; Forkel et al., 2019). However, such predictions do not fully account for connection between fire and deforestation. Consequently, we still do not fully understand how changes in deforestation have impacted the occurrence of fire.

Large‐scale deforestation of the Amazon started in the 1970s (van Marle et al., 2017) and about 15% of the Amazon had been deforested by 2010 (Aragao et al., 2014). Deforestation rates declined from 27,772 km^2^ yr^−1^ in 2004–4,571 km^2^ yr^−1^ in 2012 an impressive 84% reduction in deforestation (PRODES, Measurement of Deforestation by Remote Sensing). In, 2009, Brazil announced ambitious targets to reduce deforestation under the National Policy on Climate Change, which committed to reducing the deforestation rate in the Brazilian Amazonia by 80% by 2020 compared to a 1996–2005 baseline (Junior et al., 2020). However, since 2012, the rate of deforestation across the Brazilian Amazon has increased by a factor of two, reaching 10,129 km^2^ yr^−1^ in 2019 and 10,851 km^2^ yr^−1^ in 2020 (PRODES) (Barlow et al., 2019). By 2020, the deforestation rate was the highest rate in the last decade and 182% higher than Brazil's target (Junior et al., 2020).

The impacts of increased deforestation on the occurrence and frequency of fire have not been fully assessed and the implications of not meeting deforestation targets on fire are not known. Jones et al. (2022) found that trends in burned area in tropical forest regions, including the Amazon, were more closely related to deforestation trends compared to trends in fire weather. Increased fire in the Amazon during 2019 (Barlow et al., 2019) caused widespread media coverage (De Oliveira Andrade, 2019) but the causes are not fully understood. Kelley et al. (2021) used a Bayesian inference method to show a low meteorological influence in the enhanced 2019 Amazon fires, suggesting socio‐economic factors were the main cause of increased fire. To further explore the relationship between climate, deforestation and fire we combined spatially gridded observations of climate, land cover, and deforestation with machine learning techniques to predict monthly fire count from 2003 to 2020 across the Brazilian Amazon Biome (BAB). We used the model to make a first detailed assessment of the impact of increased deforestation on fire. Next we assessed the implications of not meeting deforestation targets on the occurrence of fire. Finally, we combined our fire predictions with a chemistry‐climate model and exposure‐response associations to predict the impacts of different deforestation scenarios on air quality and human health.

## 2 Methods

### 2.1 Feature Data for Machine Learning

Feature data used in machine learning models include: data on active fire count, deforestation rate, climate (precipitation, temperature, and leaf area index), and land‐cover. Before model training each dataset was masked to the Brazilian Amazon biome (Figure S1 in Supporting Information S1) then re‐sampled from its native spatial resolutions to a 0.25° regular grid (approximately 27–28 km at the equator). This allowed for faster and efficient model training and matched the spatial resolution of the WRF‐Chem model. Finally, all datasets were vectorized (tabulated) and placed in a single data frame for model training (see Data availability).

### 2.2 Active Fire Count Data

Daily active fire counts were taken from the MCD14DL MODIS product on board Terra and Aqua satellites (Giglio et al., 2003, 2006). Fire count data is provided as point vectors being the centroid of a 1 km by 1 km pixel in which one or more fires were detected. Fire count data is acquired continuously providing global coverage every 1–2 days. The overpass time of Terra is approximately 10:30 am and 10:30 pm each day (local time), while the overpass time of Aqua is approximately 1:30 pm and 1:30 am.

We obtained fire count data for the period from 1 January2,003 to 31 December 2020 from the Fire Information for Resource Management System (FIRMS) platform [https://firms.modaps.eosdis.nasa.gov/download/, last accessed 14.04.2021]. We removed persistent land sources of fire whose characteristics do not represent that of vegetation fires, such as gas flaring locations. Additionally, we removed fire counts with a confidence interval less than 66% leaving only high confidence active fires for analysis. Daily fire counts were summed to a monthly resolution. Due to the relatively coarse spatial resolution of MODIS, we were unable account for different fire types such as understorey fires (Berenguer et al., 2021).

### 2.3 Deforestation Data

We used annual deforestation areas from the Brazilian government's satellite monitoring program, PRODES (Program to Calculate Deforestation in the Amazon) (Assis et al., 2019). PRODES captures new, clear‐cut deforestation of primary old‐growth forests larger than 6.25 ha based on Landsat (30 m spatial resolution), Sentinel 2 (10–20 m), and CBERS 4 (10–20 m) satellites. Within a given year (reference year), PRODES monitoring captures deforestation area spanning August 1st of the previous year to July 31st in the reference year. For example, PRODES deforestation in 2019 would include all deforestation spanning 1 August 2018 to 31 July 2019. This monitoring window in the middle of the dry season, allows for better image collection since the forest is less likely to be covered by clouds, as well as coinciding with typical clearing cycles in the region. Because PRODES data represents deforestation of primary old growth forests, deforestation of secondary forest dynamics are excluded, as well as forest disturbances due to forest degradation. PRODES areas in the form of georeferenced polygons were taken from two separate sources: Global Forest Watch (GFW) for 2001 to 2007, and the National Institute for Space Research (INPE) TerraBrasilis platform (Assis et al., 2019) for 2008 to 2019. Deforestation for year 2020 were taken from TerraBrasilis based on 102 priority scenes published as of late November 2020. Older data collected through GFW use georeferencing methods in the Landsat 5/7 scenes compared to orthorectified images from Landsat 8 for newly processed data collected from TerraBrasilis. Differences between GFW data (Landsat 5/7 scenes) result in small displacements of between 60 and 90 m when compared to Landsat 8 scenes, however, these displacements were not relevant for the purpose of our analyses.

### 2.4 Precipitation Data

We used precipitation data from Climate Hazards Group InfraRed Precipitation with Stations (CHIRPS) version 2.0 (Funk et al., 2014). The CHIRPS dataset is a blended rainfall product combining a 5‐year precipitation climatology, satellite observations, atmospheric model simulated rainfall fields, and in situ observations from gauge stations. Quasi‐global gridded products are available from 1981 to near‐present at 0.05° spatial resolution (∼5.3 km) (Funk et al., 2014). Daily data spanning the period 1 January 2002 to 31 December 2020 was averaged to monthly data.

### 2.5 Land Surface Temperature Data

We used monthly mean day‐time land surface temperature (LST) from MODIS MYD11C3 (LST) on board the Aqua satellite (Wan et al., 2015) for the period 1 January 2003 to 31 December 2020. Aqua has an overpass time of approximately 1:30 pm (LT), which is close to the afternoon peak in fire activity (GIGLIO, 2007). Monthly mean LST is derived by averaging daily files on a 0.05° resolution grid.

### 2.6 Leaf Area Index Data

We used leaf area index (LAI) from MODIS collection 6 LAI (MOD15A2H) (Myneni, 2015). LAI (m^2^/m^2^) is defined as the one‐sided green leaf area per unit ground area in broadleaf canopies. LAI is calculated from MODIS reflectances and ancillary data on surface characteristics such as land cover type. We averaged 8‐day to monthly mean data after applying quality control filtering to remove cloud‐contaminated pixels.

### 2.7 Land Cover Data

Land cover data was provided by the MapBiomas project (Mapbiomas, 2020), which provides annual land cover characteristics a on 30 m grid for the period 1985 to 2019. We used data spanning years 2003–2019, using 2019 land cover for the year 2020. We identified three land cover types: pasture, cropland and savannah or.

### 2.8 Machine Learning Simulations

We used machine learning (ML) to predict gridded total monthly fire counts at 0.25° × 0.25° resolution across the Brazilian Amazon biome from 2003 to 2020 using various climate, land cover, and deforestation features (Table 1). In each simulation, three supervised ML models were used: a random forest (RF) (Breiman, 2001), a gradient boosting decision tree algorithm Xgboost (XGB) (Chen & Guestrin, 2016), and a Neural Network (NN) (Howard & Gugger, 2020). Model architectures and hyperparameters are briefly described in Tables S1–S3 in Supporting Information S1 with code for each model provided in Supporting Information S1. Hyperparameter selection for each model was based on results from a 5‐fold cross validation grid search approach. Test data was withheld for a specific year of interest and used to predict monthly fire count at the grid level for that year, while data from all other years were used for model training and validation. Under historical fire count prediction, prediction on test data were run chronologically; such that data for year 2003 was withheld as test data and data for years 2004–2020 was used as training and validation datasets. This process was repeated each year in the study period, so that the predictions for each year represented data in the test dataset. For 2020 fire count prediction, test dataset comprised data in 2020 with training and validation datasets making up data from 2003 to 2019. We used a 5‐fold cross validation approach for training and validation such that all data could be used for both training and validation. We averaged test dataset predictions (specific year of interest) across folds, as well as across the different models in that model combination which can improve predictive performance compared to predictions from any single model (Erickson et al., 2020). We found the average of predictions from models XGB and NN outperformed any single model or other combination of models (Table S4 in Supporting Information S1), so we used the average predictions from these two models. Results for individual models are reported in Supporting Information S1. Permutation feature importance was also performed using all models and model combinations to show the difference across models in terms of which features were perceived to be most important for fire count prediction. In addition, we incremented surface temperature (+0.1 K to +1K), precipitation (−1% to −10%), deforestation (+1% to +50%) in all grid cells individually across all years to assess model (NN and XGB) sensitivity to predicted fire count. The calculated average across all years was then used to show the overall prediction sensitivity to individual changes in these three features.

**Table 1:** List of Features Used in This Analysis

Feature	Units	Description	Data source
Fires	Count	Monthly total number of active fires (Jan 2003 to Dec 2020).	MODIS (MCD14DL)
Deforestation	km^2^	Total deforestation area in reference year and the preceding 2 years, such that deforestation in reference year 2020 would be total deforestation from 2018 to 2020.	PRODES
Precipitation	mm	Monthly mean precipitation (Jan 2003 to Dec 2020).	CHIRPS
Previous precipitation	mm	Total precipitation in the preceding 6 months (Jul 2002 to Dec 2020). Used to account for possible legacy effect on fires (Butt et al., 2021)	CHIRPS
Surface temperature	Degrees Celsius	Monthly mean land surface temperature.	MODIS Aqua (MYD11C3)
LAI	Area/area	Mean LAI in the preceding 12 months (Jan 2002 to Dec 2020).	MODIS (MOD15A2H)
Pasture fraction	%	Grid fraction of pasture (2003–2019).	MapBiomas
Cropland fraction	%	Grid fraction of cropland (2003–2019).	MapBiomas
Savannah fraction	%	Grid fraction of Savannah (2003–2019).	MapBiomas

*Note.* We developed models to simulate monthly fire count based on a range of variables listed in the table. All features are gridded variables at a 0.25° spatial resolution grid.

We predicted historical fire count under four different simulations (Table 2). The first simulation (Sim_Clim) used only climate features (temperature, precipitation). The second simulation (Sim_Clim + LU) used both climate and land‐use features. The third simulation included both climate and deforestation features, but did not include land‐cover. The final simulation (Sim_Clim + LU + Def) used climate, land‐cover, and deforestation features. The separation of climate, land‐cover, and deforestation features under these four simulations allows us to isolate the role of deforestation on fire activity.

**Table 2:** Description of the Four Simulations Used for Predicting Historical Fire Count

	Simulation			
	Sim_Clim	Sim_Clim + LU	Sim_Clim + Def	Sim_Clim + LU + Def
Climate	Yes	Yes	Yes	Yes
Land cover	No	Yes	No	Yes
Deforestation	No	No	Yes	Yes

To estimate the sensitivity of fires to deforestation, we predicted fire count from 2013 to 2020 under a range of scenarios using the most realistic (Sim_Clim + LU + Def) simulation. The control scenario used observed climate and observed deforestation. The average climate scenario applied a climatological monthly average of climate features over the period 2003 to 2020 with observed deforestation. The minimum deforestation scenario applied observed climate with the deforestation observed in 2012, which was the minimum deforestation from 2003 to 2020. The target deforestation scenario applied observed climate with a target deforestation of 3,283.6 km^2^ yr^−1^ for each year from 2013 to 2020, which was calculated by reducing the Brazilian government's target deforestation of 3,925 km^2^ yr^−1^ for the Brazilian Legal Amazon (BLA) by 16.34%, the difference in area between the BAB and the BLA. In the deforestation scenarios we scaled observed deforestation to the annual total across the BAB, so that the spatial pattern of deforestation was retained.

### 2.9 WRF‐Chem Regional Climate‐Chemistry Model

We used the Weather Research and Forecasting Model coupled to Chemistry (WRF–Chem) version 4.0.0 (Grell et al., 2005) to simulate ambient particulate matter concentrations in 2020 under the different deforestation scenarios. WRF‐Chem has been used previously to simulate the impacts of biomass burning in the Amazon (Butt et al., 2020, 2021; Vara‐Vela et al., 2021). The model domain included most of South America (Figure S1 in Supporting Information S1) with a horizontal resolution of 30 km, with 33 vertical levels extending from the surface to 10 hPa. Gas‐phase chemistry is calculated using the extended Model for Ozone and Related Chemical Tracers, version 4 (MOZART‐4) (Emmons et al., 2010; Knote et al., 2014). Aerosol chemistry and microphysics is simulated using Simulating Aerosol Interaction and Chemistry (MOSAIC) with aqueous chemistry and four sectional discrete aerosol size bins: 0.039–0.156 μm, 0.156–0.625 μm, 0.625–2.5 μm, 2.5–10 μm (Hodzic & Knote, 2014; Zaveri et al., 2008). A volatility basis set represents secondary organic aerosol (SOA) formation (Knote et al., 2015). Microphysics is simulated using the Morrison 2‐moment scheme (Morrison et al., 2009), and the Grell 3‐D parameterization is used for simulating convection (Grell & Dévényi, 2002). Initial and boundary chemistry and aerosol conditions were taken from 6‐hourly simulation data from Whole Atmosphere Community Climate Model (WACCM) (Gettelman et al., 2019), while initial and boundary meteorological conditions were taken from the European Center for Medium–Range Weather Forecasts (ECMWF) ERA5 global reanalysis (Hersbach et al., 2020). During WRF‐Chem simulations, we nudged the meteorological components, horizontal and vertical wind, potential temperature and water vapor mixing ratio, to ERA5 reanalysis in all model levels above the boundary layer (BL) over 6 hr. Details of the WRF‐Chem setup used in this study are shown in Table S5 in Supporting Information S1.

Anthropogenic emissions were taken from the Emission Database for Global Atmospheric Research with Task Force on Hemispheric Transport of Air Pollution (EDGAR‐HTAP) version 2.2 for the year 2010 at 0.1° × 0.1° horizontal resolution (Janssens‐Maenhout et al., 2015), while biogenic volatile organic compound (VOC) and were calculated online by the Model of Emissions of Gases and Aerosol from Nature (MEGAN) (Guenther et al., 2006).

We used emissions for landscape fires from the Fire Inventory from NCAR (FINN) (Wiedinmyer et al., 2011) version 1.5. Daily FINN emissions are estimated on a 1 km^2^ grid based on the location and timing of active fires taken from MODIS Fire and Thermal Anomalies Product (Giglio et al., 2003). Each fire count is assigned a burned area of 0.75 km^2^ for grassland and savannah and 1 km^2^ for other land covers. In WRF‐Chem, FINN emissions were emitted using a diurnal cycle that peaks in the early afternoon (local‐time) based on Giglio (2007) and are injected evenly throughout the BL, as supported by fire emission plume heights over the Amazon (Marenco et al., 2016).

Annual WRF‐Chem model simulations were conducted for the year 2020, with 1 month spin‐up. We conducted three simulations with different fire emissions: (a) a control simulation in 2020 using default fire emissions from FINN, (b) scaled 2020 FINN emissions under the period minimum deforestation scenario, and (c) scaled 2020 FINN emissions under the Brazilian government's deforestation target scenario. The difference between simulations (a) and (b) and (a) and (c) were used to quantify the impact of different deforestation scenarios on regional air quality and human health in 2020.

Based on the strong relationship between fire counts and emissions (Butt et al., 2021), we used changes in predicted fire count from our deforestation scenarios to scale default FINN emissions (*FINN*_*control*_) according to:

(1) $${\mathrm{FINN}}_{\mathrm{scaled}}=\left(\begin{array}{c} \frac{\mathrm{ML}\_\mathrm{scenario}}{\mathrm{ML}\_\mathrm{control}} \end{array}\right)\times {\mathrm{FINN}}_{\mathrm{control}}$$

where *FINN*_*scaled*_ is the FINN emissions under the deforestation scenario, *ML*_*control*_ is predicted monthly fire count in 2020 under the control scenario, and *ML*_*scenario*_ is the predicted fire count in 2020 under the deforestation scenario. Reduced deforestation scenarios show reduced annual predicted fire count (Figure S2 in Supporting Information S1) and organic carbon (OC) emissions (Figure S3 in Supporting Information S1).

### 2.10 Health Impact Assessment

We quantified the disease burden due to long‐term exposure to ambient air pollution using simulated annual‐mean PM_2.5_ concentrations under different deforestation scenarios. Fires in the Amazon exhibit low interannual variability as a result of widespread and routine human‐induced burning (Giglio et al., 2013) that has been occurring since large‐scale deforestation began in the late 1980's (van Marle et al., 2017). Our focus on long‐term PM_2.5_ exposure impacts is therefore justified because populations across the Amazon region have been exposed to PM_2.5_ from fires consistently for more than 30 years.

The disease burden is estimated using relative risk (RR) estimates of disease outcomes from the Global Exposure Mortality Model (GEMM) exposure‐response function (Burnett et al., 2018). Disease outcomes include specific non–accidental mortality from non‐communicable diseases (NCD) and lower respiratory infections (LRI), with age–specific modifiers for adults over 25 years of age in 5–years intervals. The function has a mean, lower, and upper uncertainty interval with a theoretical minimum‐risk exposure level of 2.4 μg m^−3^ under which no excess health risk is assumed. Due to a lack of associations among epidemiological studies (e.g., Burnett et al., 2014; Burnett et al., 2018), the GEMM treats all PM_2.5_ as equally toxic regardless of source, shape, or chemical composition.

Excess premature mortality (*MORT*) in each age group was calculated using:

(2) $$MORT=P\times M_{BaseRate}\left(1-1/RR_{\mathrm{EXP}}\right)$$

where *P* is the exposed population and *M*_*BaseRate*_ is the baseline mortality rate for disease outcome in the age group. Population count are from the United Nations adjusted Gridded Population of the World dataset (Version 4, Revision 11, at 15 arc−minute resolution) for the year 2020 (CIESIN, 2018), while population age composition were taken from the GBD2017 for 2015 for early–neonatal (0–6 days), late–neonatal (7–27 days), post–neonatal (8–364 days), 1–4 years, 5–95 years in 5–years intervals, and 95 years plus (Roth et al., 2018). Country‐level baseline mortality rate for each health outcome (NCD: group category B and LRI: specific category A.2.2) were taken from GBD2017 (Roth et al., 2018).The effect of chronic exposure to air pollution is known to be significantly different for morbidity and mortality regarding cardiovascular outcomes (ischemic heart disease, IHD and stroke, STR) (Cohen et al., 2017). We therefore calculated years lived with disability (YLD) using RR_adjusted_ based on:

(3) $$RR_{\mathrm{EXP},adjusted}=ratio\times RR_{\mathrm{EXP}}-ratio+1$$

applying a ratio of 0.141 for IHD and 0.553 for STR from the GBD2016 (Cohen et al., 2017).

Modifying Equation 2 from calculating premature mortality, we then estimated years of life lost (YLL) and YLD per health outcome and age bracket using country‐level baseline rates of YLL and YLD from GBD2017 (Roth et al., 2018). As a final step, we estimated disability–adjusted life years (DALYs), that is, the total loss of healthy life, as the total of YLL and YLD:

(4) DALYs=YLL+YLD

Final health burden assessment included total burden values of premature excess mortality and morbidity (deaths and DALYS) and rates of deaths and DALYS per 100,000 population. Mean estimates were quantified in addition to upper and lower uncertainty intervals corresponding to the GEMM function. Shapefiles were then used to aggregate results at the country level (Hijmans et al., 2012).

Health burden impacts due to ambient PM_2.5_ depend non‐linearly on long‐term exposure, with impacts starting to saturate at high PM_2.5_ concentrations (Burnett et al., 2014, 2018). Using the “subtraction” method (Conibear et al., 2018; Kodros et al., 2016), we estimate the averted health burden in 2020 based on WRF‐Chem simulated PM_2.5_ concentrations under different deforestation scenarios relative to a control scenario. We use the subtraction method because this method provides the averted health burden due to the reduction in fire emissions associated with a reduction in deforestation rather an attributing the health burden to fires in general.

## 3 Results and Discussion

### 3.1 Fires and Deforestation

**Figure 1:**
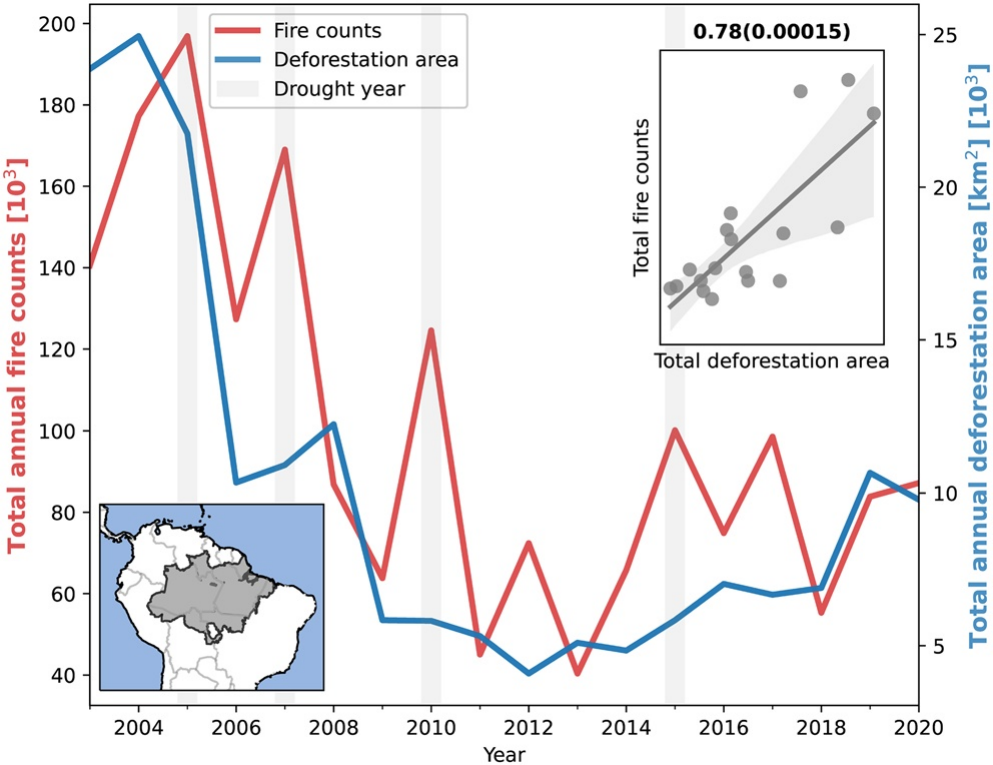
Annual time‐series of total fire count and total deforestation area (increments) across the Brazilian Amazon biome (BAB). Drought years indicated by gray shading. Pearson's correlation coefficient (and p‐value) is included as part of the inserted scatter plot.

Figure 1 shows the relationship between annual fire count and deforestation area from 2003 to 2020 across the Brazilian Amazon. Deforestation area and fire count are consistent with those reported previously using the same datasets (Libonati et al., 2021; Silveira et al., 2020). The connection between climate and fire is apparent with higher fire count occurring during drought years (2005, 2007, 2010, and 2015). Annual fire count and deforestation are also strongly correlated (*r* = 0.78, *p* < 0.01) and previous suggestions that fire and deforestation have decoupled (Aragão et al., 2018) are less clear in this longer time series. Declines in fires follow declines in deforestation during 2001–2014, as reported previously (Reddington et al., 2015). Since the minimum in deforestation and fire count observed in 2012–2013, both fire count and deforestation have more than doubled (Arãgao et al., 2008; Aragão et al., 2018; Barlow et al., 2019). Analysis of monthly deforestation and fire count shows that deforestation peaks at the end of the wet season, before the dry season peak in fires, suggesting that within a specific year, fires are not the dominant cause of deforestation (Butt et al., 2021).

### 3.2 Historical Fire Prediction

**Figure 2:**
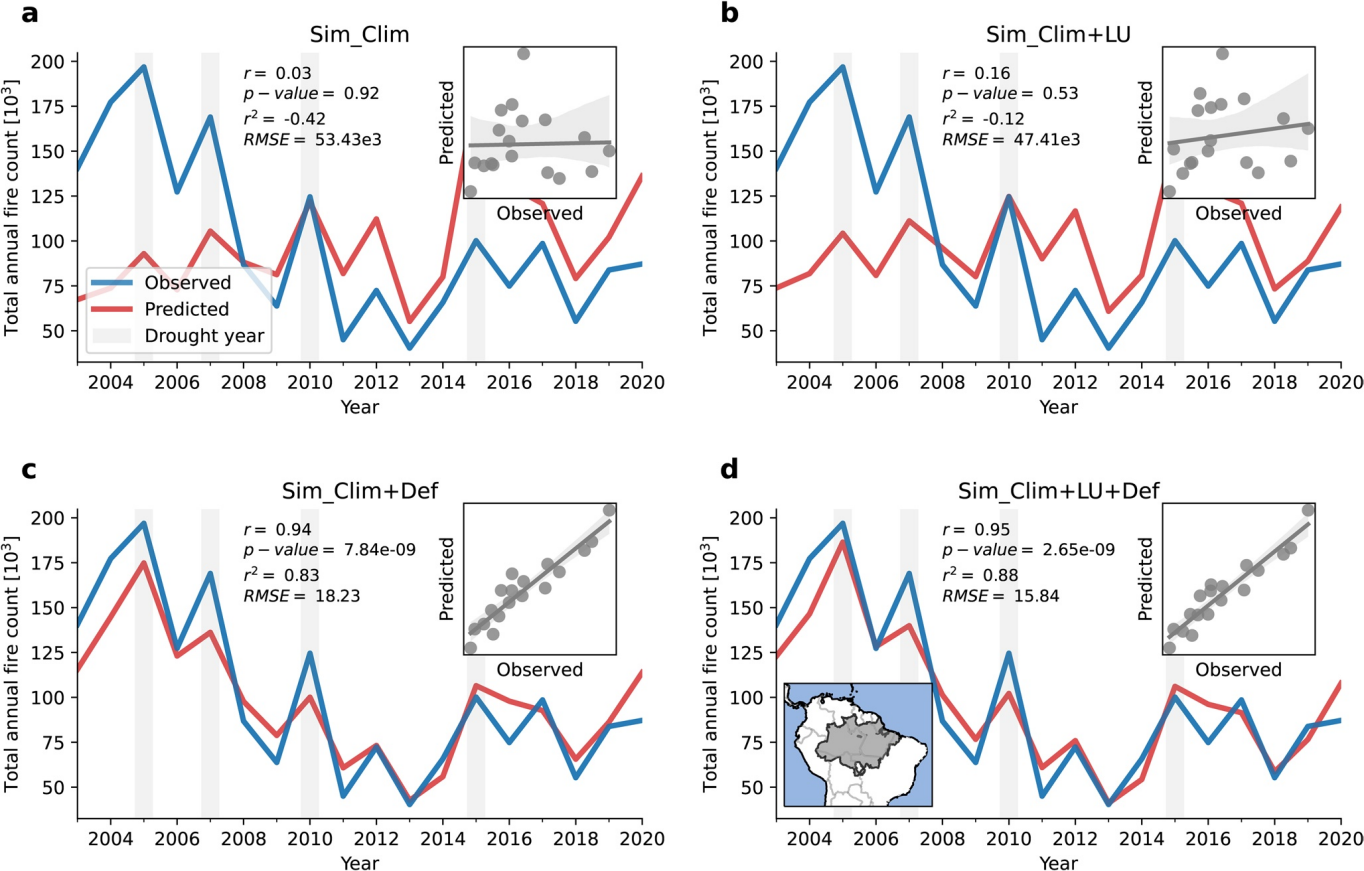
Annual observed (blue line) and predicted (red line) fire count across the Brazilian Amazon biome (BAB) under simulations using (a) only climate features: Sim_Clim, (b) climate and land‐use cover features: Sim_Clim + LU, (c) climate and deforestation features: Sim_Clim + Def, and (d) climate, land‐use, and deforestation features: Sim_Clim + LU + Def. Predictions are shown for the model combination XGBoost (XGB) and artificial neural network (NN) because this was found produce the lowest *RMSE* for any model or model combination under the most realistic simulation (Sim_Clim + LU + Def) (Table S4 in Supporting Information S1). Pearson's correlation coefficient (*r*), p‐value, coefficient of determination (*r* ^*2*^), and root mean squared error (*RMSE*) are reported in each panel.

Figure 2 shows annual total observed and predicted fire count from 2003 to 2020. When the model is trained only using climate‐related features, fire count predictions are relatively poor (Figure 2a; coefficient of determination [*r* ^2^] = −0.42, p‐value [*p*] = 0.92, root mean squared error [*RMSE*] = 53.43e3). The negative *r* ^2^ under this model signifies a poorer model prediction than if the mean of the target observed fire count had been used. The model performance improves marginally if land‐cover features are also included in model training (Figure 2b; *r* ^*2*^ *=* −0.12, *p =* 0.53, *RMSE =* 47.41e3). Including annual deforestation in addition to climate greatly improved predicted annual fire count (Figure 2c; *r* ^*2*^ *=* 0.83, *p =* 7.84e − 09, *RMSE =* 18.23e3). The best prediction occurs when climate, land‐cover, and deforestation rate are used to train the model (Figure 2d: *r* ^*2*^ *=* 0.88, *p =* 2.65e−09, *RMSE =* 15.84e3). Spatially predicted fire count is also improved when deforestation features are included in model training (Figure S4 in Supporting Information S1). The tight link between annual deforestation rate and annual fire count suggests fire is largely caused by deforestation rather than legacy effects of increased susceptibility of fragmented forests to fire (Silva Junior et al., 2018).

**Figure 3:**
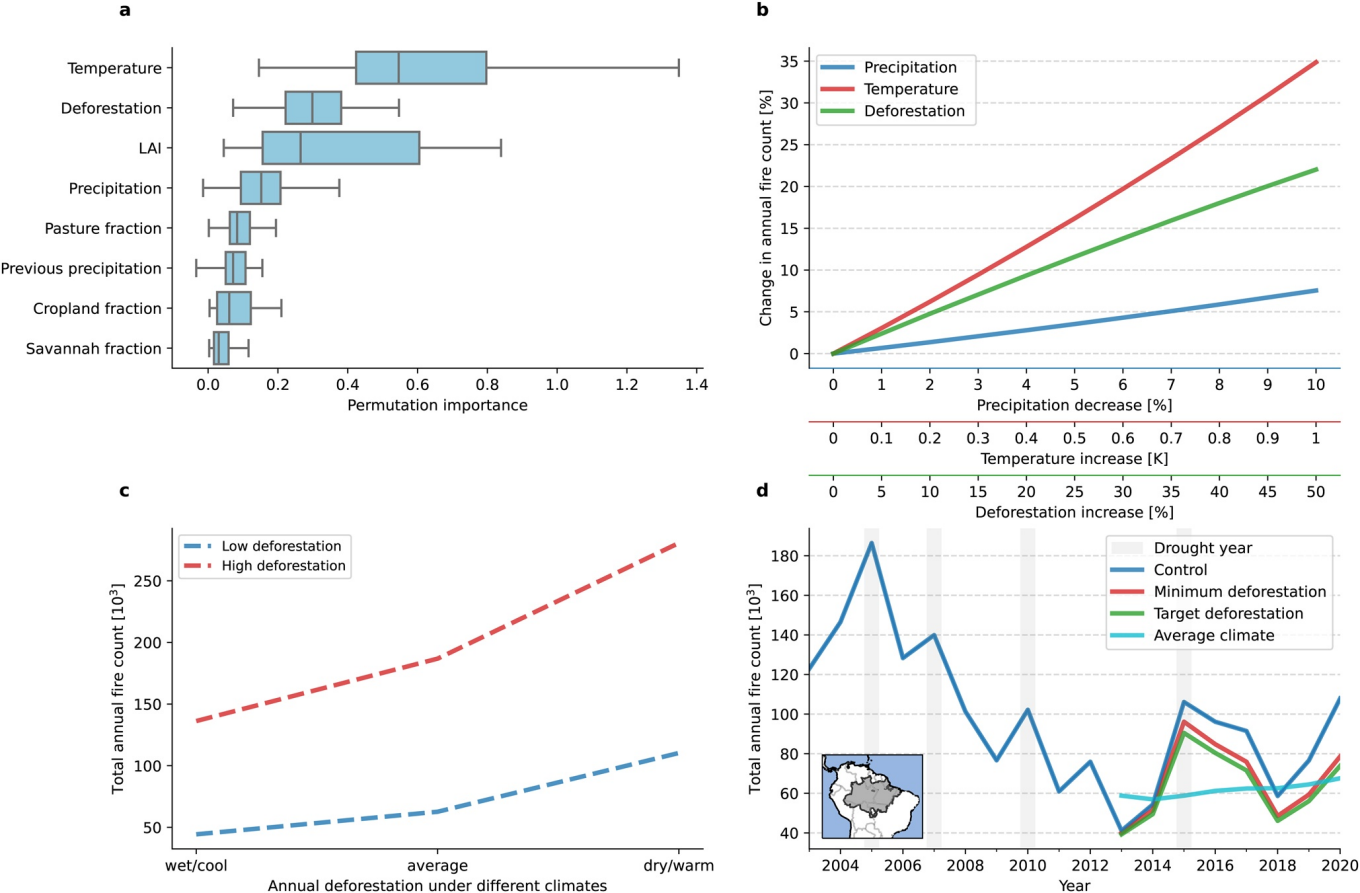
(a) Permutation importance across all years (2003–2020) representing the importance of features for fire count prediction. Boxes show quartiles of the calculated permutation importance across individual years the median of which showing 50th percentile. Calculated permutation importance is taken as an average combination of neural network (NN) and XGBoost (XGB). Permutation importance for individual models and all model combinations are shown in Figue S3 in Supporting Information S1. (b) Model (NN and XGB) prediction sensitivity showing annual change in total fires across the BAB as a function of incremental changes in surface temperature (+0.1 K to +1K), precipitation (−1% to −10%) and deforestation (+1% to +50%) calculated as an average across all years (2003–2020). (c) Annual predicted fire count for both lowest measured (low) deforestation year (2013) and highest measured (high) deforestation year (2004) under different climates: wettest and coolest (wet/cool) year (2013), driest and warmest (dry/warm) year (2015), and average climate. (d) Predicted annual total fire count for the control (observed deforestation, climate, and land‐cover for each individual years from 2003 to 2020) and the minimum deforestation (observed climate, but with deforestation area reduced to the period minimum), Brazil's deforestation target (observed climate, but with deforestation area reduced to the Brazilian government's target), and average climate (observed deforestation area, but with average of climate features for years 2003–2020) scenarios. All scenarios used features in the Sim_Clim + LU + Def simulation (Table 2) averaged across XGB and NN models.

Predicted fire count is sensitive to temperature, deforestation rate, leaf area index (LAI) and precipitation (Figure 3a; Figure S5 in Supporting Information S1). The importance of temperature on fire prediction is consistent with previous work (Lima et al., 2018). We find that fire count increases by 3.5% for every +0.1 K increase in surface monthly temperature, by 0.8% for every percentage point reduction in monthly precipitation and by 0.44% for every percentage point increase in annual deforestation rate (Figure 3b). Deforestation impacts fire count under both cool/wet as well as dry/warm conditions (Figure 3c). In a wet and cool year, fire counts increase from about 50,000 under low deforestation to 145,000 under high deforestation (an increase of 205%). In a warm and dry year, fire count increases from about 100,000 under low deforestation to 250,000 under high deforestation (increase of about 155%). Crucially, this result suggests that reduced deforestation is likely to reduce fire risk even under warmer, drier conditions that will become more prevalent under climate change (Marengo et al., 2018).

Fire count predictions that do not include deforestation provide further information on the role of climate and land cover in decadal fire trends in the Amazon (Figures 2a and 2b). Under these predictions, annual predicted fire count increases from 2003 to 2012, opposite to the observed trend. This result is consistent with deforestation contributing to the high fire count during the early 2000s (Chen et al., 2013). The predicted positive trend in fire count in these models suggests that increased pasture fraction (Figure S6 in Supporting Information S1), warming and increased drought frequency have contributed to increased fire. We can use the model sensitivity to temperature to estimate the 0.6–0.7 K warming of the Amazon over the past 40 years (Marengo et al., 2018) is likely to have contributed to a 21%–25% increase in fire count. Our findings show that good performance in predicting the variability and trend in fire count over the study period is only achieved when accounting for deforestation.

### 3.3 Fires Under Different Deforestation Scenarios

Figure 3d compares annual predicted fire count under different deforestation scenarios. The increase in deforestation between 2012 and 2020 has caused a 43% increase in fire count from the fire minimum year 2013–2020. Under Brazil's target deforestation rate we predict fire count would have been 32% lower in 2020 than observed (Figure 3d). The largest reductions in fire occur across the deforestation frontier (Figure S7a in Supporting Information S1) during August to October (Figure S7b, Table S6 in Supporting Information S1). The strong control of climate in the inter‐annual variability in fire count is apparent with similar variability of fire in the simulations driven by observed climate and much weaker variability in the simulation with average climate (Figure 3d).

### 3.4 Air Quality Under Reduced Deforestation

**Figure 4:**
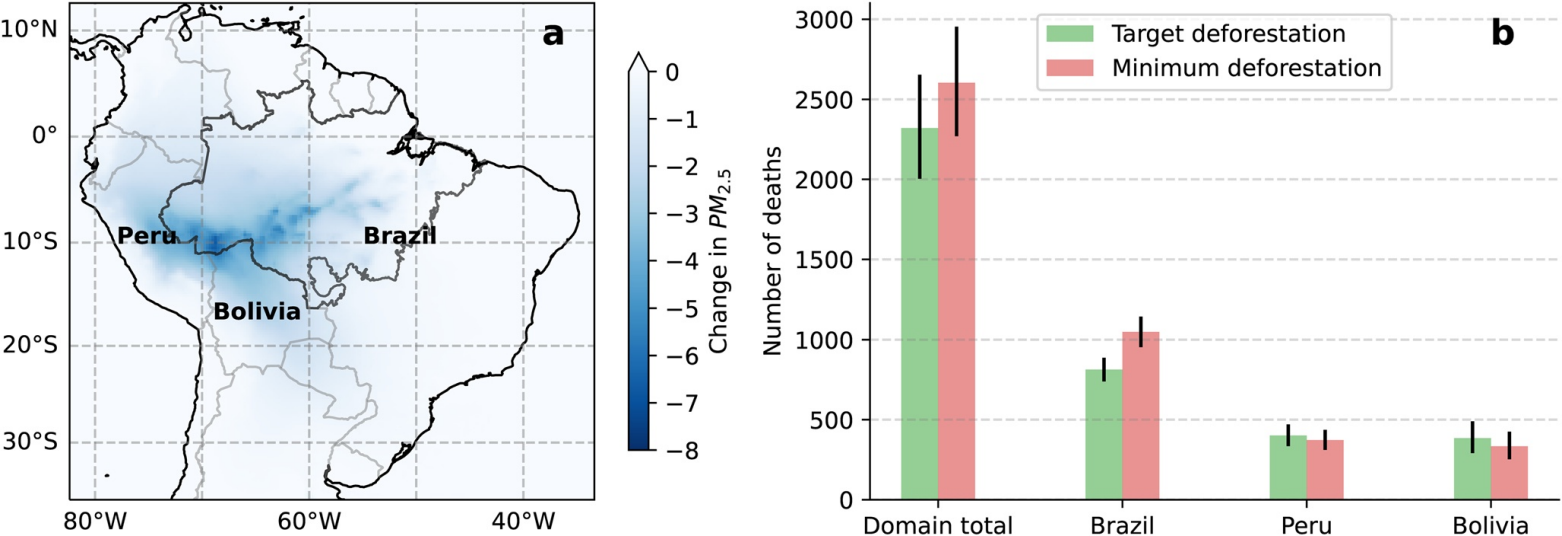
(a) Reduction in simulated annual‐mean surface ambient PM_2.5_ concentrations (μg/m^3^) in 2020 under Brazil's deforestation target under the National Policy on Climate Change relative to the control simulation (Sim_Clim + LU + Def: models XGB and NN (target – control). (b) Deaths avoided due to the reduction in fire associated PM_2.5_ under Brazil's deforestation target and minimum deforestation scenario for the total domain (all countries show in a) and separately for Brazil, Peru, and Bolivia.

Fires degrade regional air quality over the Amazon (Butt et al., 2020, 2021; Johnston et al., 2012; Lelieveld et al., 2015; Reddington et al., 2015, 2019) resulting in increased hospital admissions, adverse respiratory health outcomes and premature mortality (Do Carmo et al., 2013; Ignotti et al., 2010; Jacobson et al., 2012, 2014; Machado‐Silva et al., 2020; Smith et al., 2014). We used the Weather Research and Forecasting Model with Chemistry (WRF–Chem) to simulate annual‐mean ambient particulate matter concentrations in 2020 under the different deforestation scenarios. Under Brazil's target deforestation scenario, annual‐mean PM_2.5_ concentrations were reduced by 7 μg m^−3^ near and downwind of fire locations relative to the control simulation (Figure 4a), with population‐weighted PM_2.5_ concentrations being reduced by 1.3 μg m^−3^, 0.7 μg m^−3^, and 0.1 μg m^−3^ in Bolivia, Peru, and Brazil, respectively. Using simulated PM_2.5_ concentrations and epidemiological exposure‐response associations, we estimate PM_2.5_ reductions achieved under Brazil's target deforestation scenario would have resulted in 2,300 (95CI: 2,000–2,650) fewer deaths and 88,140 (95CI: 68,250–111,720) fewer disability adjusted life years (DALYs) (Figure 4b and Table 3) in 2020. Health benefits are greatest in Brazil but also extend regionally to Peru and Bolivia (Figure 4b). Our results are consistent with our previous work using non‐spatial state‐wide data on deforestation, climate and fire (Butt et al., 2021). Modeling studies have estimated that smoke from vegetation fires across the Amazon causes 7,000 to 17,000 air pollution‐related premature deaths annually (Butt et al., 2020, 2021; Johnston et al., 2012; Reddington et al., 2015). Achieving Brazil's target deforestation rate could therefore reduce the number of premature mortalities due to exposure to smoke from fires by 14%–32%. The range in the health impact due to vegetation fires are partly caused by the differences in health burden assessment methods used across these studies. Previous work has shown that reductions in deforestation and associated fires during 2001–2015 resulted in improved air quality and the prevention of 400 to 1,700 premature deaths annually across South America (Reddington et al., 2015).

**Table 3:** Domain‐Wide Avoided Health Burden Deaths and DALYs (Disability Adjusted Life Years) in 2020 Due To the Reduction in Fire Associated Annual Ambient PM_2.5_ Concentrations Under the Target and Minimum Deforestation Scenarios

Scenario	Deaths	DALYs
Target deforestation	2,320 (95CI: 2,000—2,650)	88,140 (95CI: 68,250—111,720)
Minimum deforestation	2,600 (95CI: 2,270—2,950)	99,510 (95CI: 77,590—125,550)

*Note*. Health burden estimates were calculated using epidemiological exposure‐response associations and WRF‐Chem simulated PM_2.5_ concentrations.

### 3.5 Limitations

Our study demonstrates relationships between deforestation rate, climate and fire, but does not explicitly include additional interactions and feedbacks which may further amplify these relationships. Deforestation impacts local and regional climate, causing local and regional warming (Baker & Spracklen, 2019), reduced evapotranspiration and reduced regional and downwind rainfall (Costa & Pires, 2010; Leite‐Filho et al., 2019; Spracklen et al., 2012; Staal et al., 2018). Smoke from fires results in modifications to atmospheric heating and alters cloud droplet concentrations, which may impact rainfall (Kolusu et al., 2015; Twohy et al., 2021). A combination of hotter and drier conditions caused by deforestation and smoke will further strengthen the relationship between deforestation and fire, making the Amazon increasingly prone to fire (Brando et al., 2020; Le Page et al., 2017). Southeast Amazonia, which has been subject to greater deforestation, warming, moisture stress, and fire, is now a net carbon source to the atmosphere (Gatti et al., 2021) contributing to warming at a global scale.

Our work focuses on the impacts of primary clear‐cut deforestation. Forest degradation caused by fires and selective logging (Silva Junior et al., 2021) makes tropical forests more flammable and susceptible to future fire (Barni et al., 2021; Cochrane & Laurance, 2008). Together these interactions and feedbacks are likely to amplify the connections between deforestation, climate and fire in the Amazon. Our findings show that deforestation rate strongly impacts fire count, but legacy effects of increased susceptibility of fragmented forests to fire, which we do not account for directly, likely play an important role (Silva Junior et al., 2018). Droughts spanning several months cause gradual drying of the forest canopy and increase susceptibility of forests to fire (Ray et al., 2005). The intense drought induced by the 2015/2016 El Niño was sufficiently strong to dry out primary forests and more than 1 million hectares burned (Withey et al., 2018). While we do account for total rainfall in the preceding 6 months, future work may benefit from treatment of a lagged drought feature.

Increasing the number of features can lead to collinearity between features. In our study, there is limited collinearity between features except deforestation and pasture fraction (Figure S8 in Supporting Information S1). However, we find that the inclusion of pasture fraction without deforestation results in poor fire count prediction, while the inclusion of deforestation rate without pasture fraction results in good fire count prediction (Figure 2) suggesting deforestation rate is the most important feature and this collinearity is not important.

Our estimates of the air quality benefits of reduced deforestation relies on a linear scaling between fire count and fire emissions, with this relationship confirmed by our previous work (Butt et al., 2021). FINN emissions account for different land covers and biomass loads (Wiedinmyer et al., 2011) meaning our projected emissions also include this variability. However, future work may benefit from prediction of fire emissions directly rather than fire count. We predict monthly fire count at a scale of ∼30 km. Future work needs to consider the importance of different spatial and temporal scales in these predictions (McLauchlan et al., 2020).

Our work shows how achieving deforestation targets reduces fire frequency and improves air quality resulting in reduced air pollution health impacts. However, placing our health burden estimates into context of previous work is difficult. The sensitivity of health burden estimates to chosen assessment methods, particularly the concentration exposure‐response association used, is well known (e.g., Ostro et al., 2018). We use the GEMM exposure‐response function calculated exclusively from ambient outdoor PM_2.5_ concentrations and health outcomes (Burnett et al., 2018), which is superior to older exposure‐response associations (e.g., Burnett et al., 2014; Cohen et al., 2005) used in other studies (e.g., Johnston et al., 2012; Lelieveld et al., 2015; Reddington et al., 2015). The spatial resolution of simulated PM_2.5_ will further contribute to differences across health burdens estimates. We simulate PM_2.5_ at resolution of around 30 km compared to resolutions of >100 km used by global modeling studies (Johnston et al., 2012; Lelieveld et al., 2015; Reddington et al., 2015). While employing higher spatial resolution would likely further improve simulated PM_2.5_ concentrations, such simulations are at the expense of greater computational cost.

## 4 Conclusions

We use machine learning with satellite datasets to estimate that a 1% point reduction in deforestation rate leads to a 0.44% point reduction in fire counts across the Brazilian Amazon. We show that if Brazil had achieved its Amazon deforestation target there would have been a 32% reduction in fire count in 2020 relative to the observed fire count. We combined this prediction with a regional air quality model and exposure‐response relationships to estimate that the improved air quality due to this reduction in fire would result in 2,300 fewer premature mortalities. New research is needed to understand how future fire risk varies under different scenarios of land use, deforestation rate and climate change. We estimate that warming over the Amazon over the past 40 years has already driven a 21%–25% increase in fire. Future climate change will further increase flammability of the Amazon forest highlighting the urgent need to find sustainable solutions to reduce deforestation, forest degradation and fire (Brando et al., 2020). Previous work has called for forest fire reduction to be integrated into reduced deforestation programs (Barlow et al., 2012). Our findings highlight that fire management and reduction programs must also integrate efforts to reduce deforestation. Our work demonstrates the benefits of reduced deforestation on air quality and public health across the Amazon through a reduction in fire. Brazil's past success in reducing deforestation and fire (Reddington et al., 2015) through effective environmental governance demonstrates considerable potential to be a global leader in sustainable management of tropical forests.

## Conflict of Interest

The authors declare no conflicts of interest relevant to this study.

## Supporting information

Supporting Information S1

## Data Availability

The data that support the findings of this study are available at https://doi.org/10.5518/1152. The code used to run machine learning models can also be found at the same address.
