# Supplementary material for: Achieving Brazil's Deforestation Target Will Reduce Fire and Deliver Air Quality and Public Health Benefits
Source: Earths Future. 2022 Dec 5;10(12):e2022EF003048. doi: 10.1029/2022EF003048 (PMC10078148; doi:10.1029/2022EF003048)
Supplement: Supplementary file 1 — Supporting Information S1 [file EFT2-10-0-s001.pdf]

Supporting Information for

Achieving Brazil's deforestation target will reduce fire and deliver air quality and public health benefits

Edward W. Butt[1*], Luke Conibear[1], Callum Smith[1], Jessica C. A. Baker[1], Richard Rigby[1], Christoph Knote[2], and Dominick V. Spracklen[1]

[1]School of Earth and Environment, University of Leeds, Leeds, UK

[2]Model-based Environmental Exposure Science, Faculty of Medicine, University of Augsburg, Augsburg, Germany

*Correspondence to Edward W. Butt: e.butt@leeds.ac.uk

Contents of this file

Tables: Supplementary Tables 1 to 6

Figures: Supplementary Figures 1 to 8

Supplementary Table 1: Random forest regression hyperparameter selection. Hyperparameter selection was largely chosen based on results from a 5-fold cross validation grid search approach in addition to some manual trial and error. Default values were used in cases where hyperhparameters are not listed. We used the Python package scikit-learn (v1.0.1) for the random forest model used in this study. A Jupyter notebook containing code for the random forest model used in this paper can be found at https://doi.org/10.5518/1152.

Hyperparameter	Value
**n_estimators**	200
**max_features**	0.5
**max_depth**	12
**min_samples_leaf**	30
**min_samples_split**	80
**max_samples**	500_000

Supplementary Table 2: XGBoost regression hyperparameter selection. Hyperparameter selection was largely chosen based on results from a 5-fold cross validation grid search approach in addition to some manual trial and error. Default values were used in cases where hyperhparameters are not listed. We used the Python package xgboost (v 1.5.0) for the XGBoost model used in this study. A Jupyter notebook containing code for the XGBoost model used in this paper can be found at https://doi.org/10.5518/1152.

Hyperparameter	Value
**n_estimators**	80
**learning_rate**	0.1
**max_depth**	8
**colsample_bytree**	0.8
**reg_alpha**	1.2
**reg_lambda**	1.2
**subsample**	0.9

Supplementary Table 3: Neural network architecture. Hyperparameter selection was largely chosen based on results from a 5-fold cross validation grid search approach in addition to some manual trial and error. We used the Python package fastai (v 2.5.1) for the tabular neural network model used in this study. A Jupyter notebook containing code for the neural network model used in this paper can be found at https://doi.org/10.5518/1152.

Layer type	Output Shape	Parameter number	Trainable
	5000 x *		
**BatchNorm1d**		16	True
	5000 x 1000		
**Linear**		8000	True
**ReLU**			
**BatchNorm1d**		2000	True
**Dropout**			
	5000 x 500		
**Linear**		500000	True
**ReLU**			
**BatchNorm1d**		1000	True
**Dropout**			
	5000 x 1		
**Linear**		501	True
**SigmoidRange**			

Total of parameters: 511,517
Total number of trainable parameters: 511,517
Total number of non-trainable parameters: 0

Optimizer used: Adam
Loss function: FlattenedLoss of MSELoss()
***Depends on the simulation running**

Supplementary Table 4: Average grid-level root mean squared error (RMSE) for test data across all months (2003 to 2020) under each simulation for individual models (RF (Random forest), XGB (XGBoot), and NN (Neural network)) and model combinations. Combination values are calculated as an average RMSE across models. The lowest RMSE is reported in bold for model combination XGB and NN under the most realistic simulation Sim_Clim+LU+Def.

Model	RF	XGB	NN	Combination All	Combination RF & XGB	Combination RF & NN	Combination XGB & NN
**Simulation**							
**Sim_Clim**	8.72	8.76	8.75	8.72	8.735	8.72	8.73
**Sim_Clim+LU**	8.20	8.18	8.25	8.15	8.17	8.16	8.17
**Sim_Clim+Def**	7.37	7.38	7.35	7.31	7.34	7.32	7.32
**Sim_Clim+LU+Def**	7.21	7.10	7.03	7.01	7.11	7.01	**6.99**

Supplementary Table 5: WRF-Chem model setup.

**General model setup**	
**Process**	Method
**Model domain**	-33° to -82° West, -36° to 13° North
**Time-step**	120 seconds, with Runge-Kutta 2nd and 3rd order time integration
**Horizontal**	Resolution of 30 km along a 179×189 grid
**Vertical**	33 vertical levels (surface at 10 hPa) with terrain-following hydrostatic pressure
**Microphysics scheme**	Morrison 2-moment (Morrison et al., 2009)
**Convection scheme**	Grell 3-D ensemble (Grell and Dévényi, 2002)
**Land-surface scheme**	Noah Land Surface Model (Ek et al., 2003)
**Boundary layer scheme**	Mellor-Yamada Nakanishi and Niino 2.5 (Nakanishi and Niino, 2006)
**Radiation scheme**	RRTM (Iacono et al., 2008)
**Gas-phase chemistry scheme**	MOZART-4 (Emmons et al., 2010)
**Photolysis scheme**	TUV (Tie et al., 2003)
**Aerosol scheme**	MOSAIC, 4 bin aerosol configuration
**Initial and boundary: chemistry/aerosol**	WACCM (Gettelman et al., 2019)
**Initial and boundary: meteorology**	6-hourly ERA5 (Hersbach et al., 2020)

Supplementary Table 6: Total predicted monthly fire count in 2020 for the control (Sim_Clim+LU+Def, for models XGB and NN) and three additional fire reduction scenarios, which are based on the control where total deforestation area is reduced to both the period minimum and Brazilian government's target, and using an average of climate features. Column 1 shows monthly total fire count in the control (10^3), while columns 2-4 show fire reduction scenarios: monthly fire count (10^3), absolute difference (scenario – control) and percentage change (scenario – control) / control.

		Fire reduction scenarios		
	**Control**	**Averaged climate**	**Minimum deforestation**	**Target deforestation**
**Jan**	1.08	0.90 (-0.18, -16.88)	0.94 (-0.13, -12.43)	0.93 (-0.15, -14.10)
**Feb**	1.47	1.03 (-0.44, -30.16)	1.21 (-0.26, -17.53)	1.17 (-0.30, -20.18)
**Mar**	1.92	1.06 (-0.86, -44.97)	1.40 (-0.53, -27.40)	1.32 (-0.60, -31.12)
**Apr**	0.52	0.28 (-0.24, -46.05)	0.46 (-0.06, -12.14)	0.45 (-0.07, -13.78)
**May**	0.48	0.41 (-0.07, -14.29)	0.45 (-0.04, -7.35)	0.44 (-0.04, -8.59)
**Jun**	1.67	1.24 (-0.43, -25.70)	1.38 (-0.29, -17.49)	1.33 (-0.34, -20.10)
**Jul**	7.17	3.94 (-3.23, -45.04)	5.29 (-1.88, -26.28)	4.97 (-2.20, -30.71)
**Aug**	30.02	17.63 (-12.39, -41.28)	20.45 (-9.57, -31.88)	18.86 (-11.15, -37.15)
**Sep**	36.61	22.37 (-14.24, -38.90)	26.10 (-10.51, -28.71)	24.48 (-12.13, -33.14)
**Oct**	21.55	10.95 (-10.60, -49.17)	16.21 (-5.34, -24.79)	15.33 (-6.22, -28.88)
**Nov**	3.47	5.99 (2.52, 72.65)	2.99 (-0.48, -13.81)	2.90 (-0.57, -16.51)
**Dec**	1.92	1.79 (-0.12, -6.44)	1.72 (-0.19, -10.14)	1.68 (-0.24, -12.65)
**Annual**	107.88	67.58 (-40.29, -37.35)	78.58 (-29.29, -27.15)	73.86 (-34.02, -31.53)

Supplementary Figures

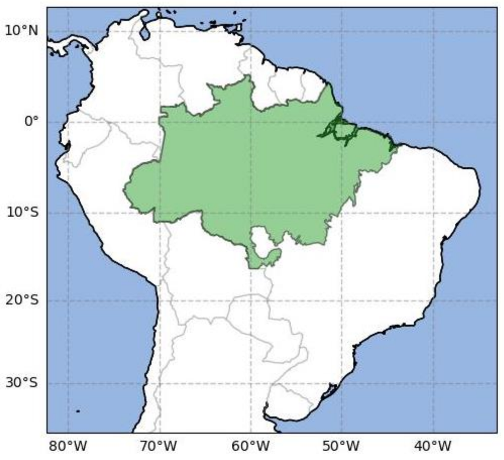

Supplementary Figure 1: WRF-Chem climate-chemistry model domain used in this. Green polygon represents the boundary of the Brazilian Amazon Biome (BAB) and region of machine learning fire count prediction

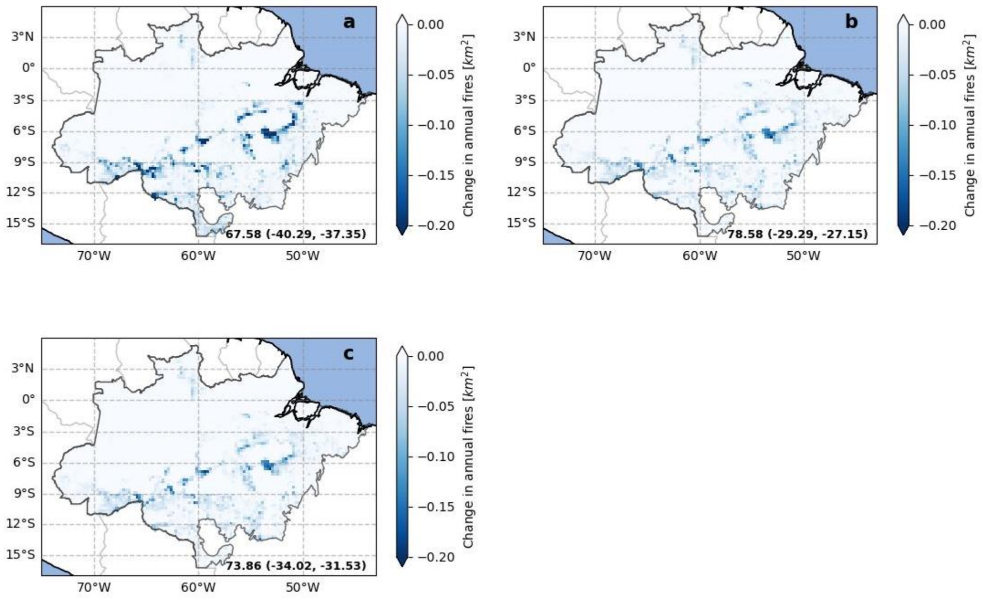

Supplementary Figure 2: Absolute difference in annual total predicted fire count (fires per km^2) in 2020 under fire reduction scenarios relative to the control simulation (Sim_Clim+LU+Def: models XGB and NN (scenario – control) (**a**) average climate, (**b**) minimum deforestation, and (**c**) target deforestation. Bold numbers reported in panels show total predicted fire count in scenario followed by annual total absolute difference (scenario – control) and percentage change (scenario – control) / control.

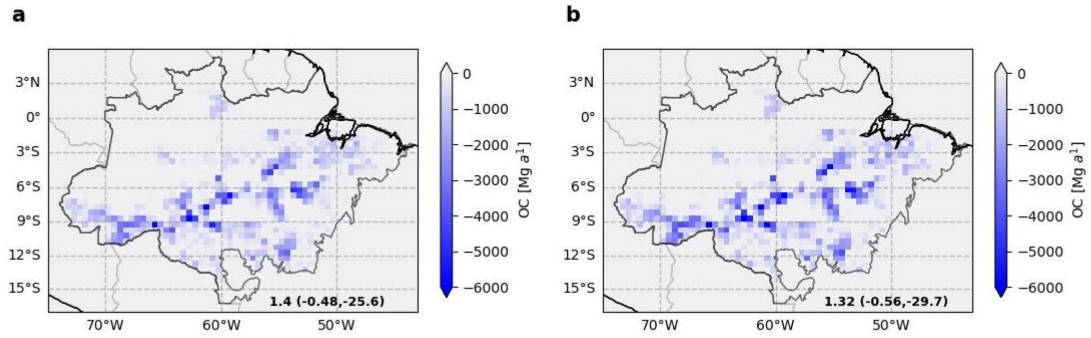

Supplementary Figure 3: Absolute difference in 2020 annual organic carbon (OC) fire emissions from FINNv1.5 under (**a**) minimum deforestation scenario and (**b**) target deforestation scenario. Emissions have been gridded to 0.5° × 0.5° resolution. Numbers reported in panel **a**-**b** show biome total annual fire emissions (Tg) in scenario followed by the absolute difference (scenario – control) and percentage change (scenario – control) / control.

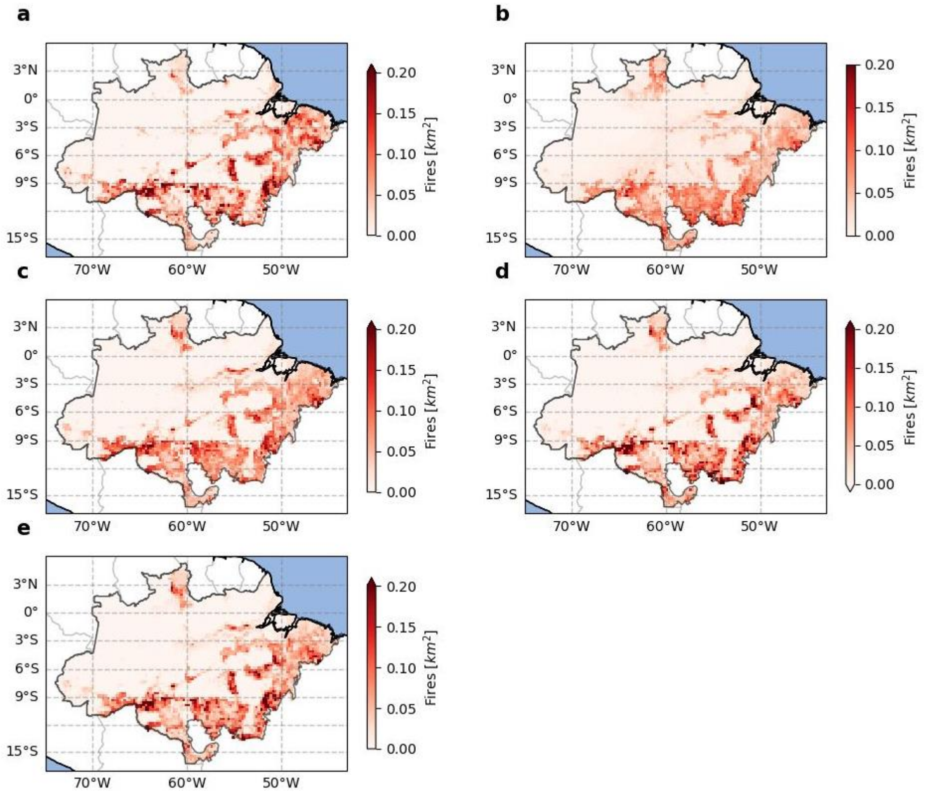

Supplementary Figure 4: Period average (2003 to 2020) annual mean (a) observed active fire count (fires per km^2) from MODIS MCD14DL and simulated fire count from (**b**) Sim_Clim, (**c**) Sim_Clim+LU, (**d**) Sim_Clim+Def, (**e**) Sim_Clim+LU+Def. Simulations including deforestation (**d**-**e**) generally better resolve fire 'hotspot' locations due to deforestation compared to simulations that do not include deforestation in training (**b**-**c**).

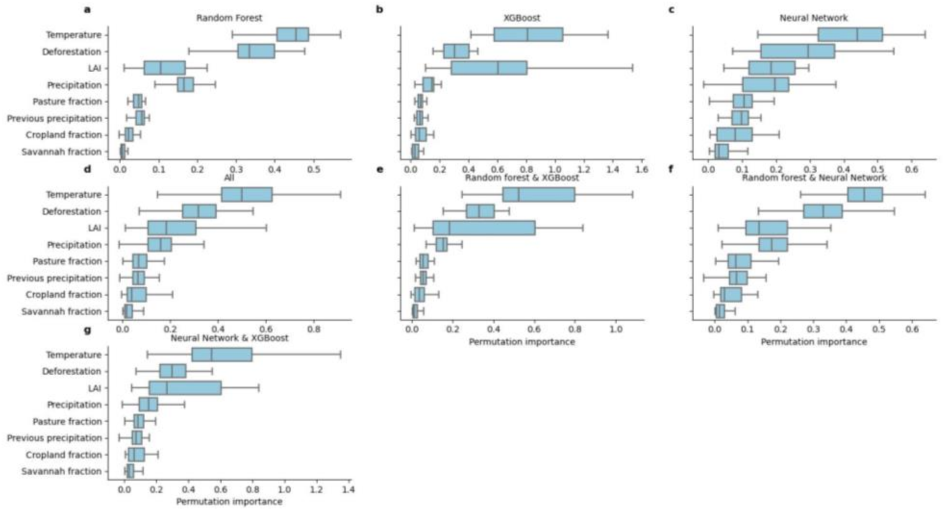

Supplementary Figure 5: Permutation importance for all years (2003 to 2020) representing the importance of features for fire count prediction for individual models and model combinations. Boxes show quartiles of the calculated permutation importance across individual years the median of which showing 50th percentile. (**a**) Random forest, (**b**) XGBoost, (**c**) Neural network, (**d**) average of all models, (**e**) average of random forest and XGBoost, (**f**), average of random forest and neural network, and (**g**) average of neural network and XGBoost.

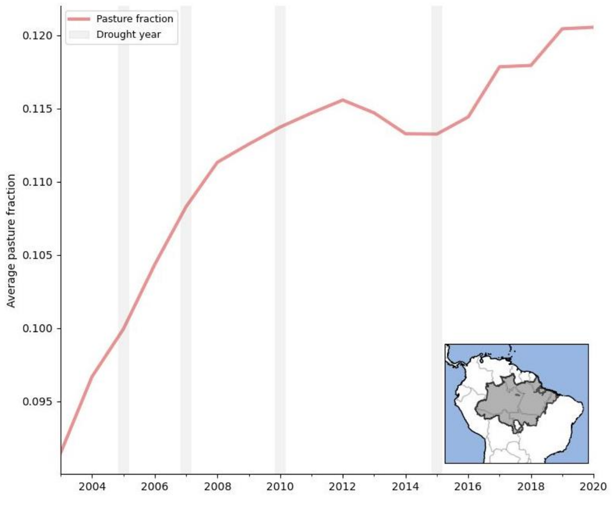

Supplementary Figure 6: Time-series of annual mean fraction of pasture land in the Brazillain Amazon biome during the period 2003 to 2020.

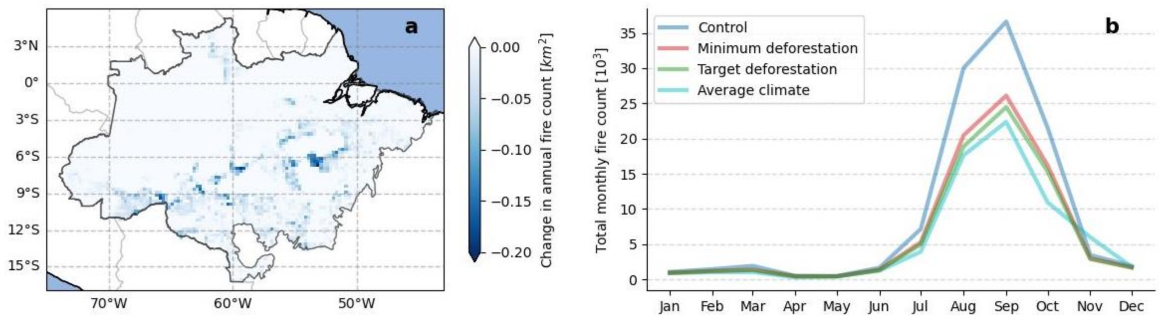

Supplementary Figure 7: Reduction in annual total predicted fire count (fires per km^2) in 2020 under the target deforestation scenario relative to the control simulation (Sim_Clim+LU+Def: models XGB and NN (target – control) (**a**) target deforestation. (**b**) Predicted monthly total fire count for the control (observed deforestation, climate, and land-cover for each individual in 2020) and the minimum deforestation (observed climate in 2020, but with deforestation area reduced to the period minimum), Brazil's deforestation target (observed climate in 2020, but with deforestation area reduced to the Brazilian government's target), and average climate (observed deforestation area in 2020, but with average of climate features for years from 2013 to 2020) scenarios. All scenarios used features in the Sim_Clim+LU+Def simulation (Table 2) averaged across XGB and NN models.

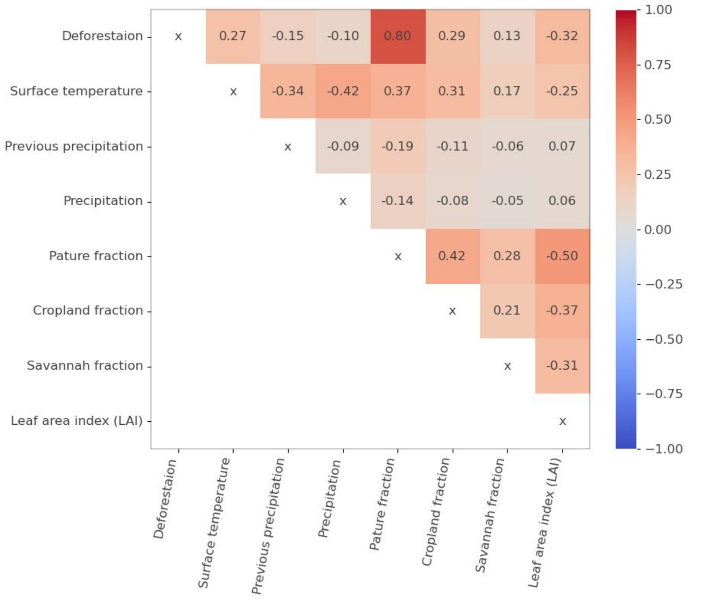

Supplementary Figure 8: Spearman's rank-order correlation matrix between all pairs of model features**.**

References from the Supporting Information

Ek, M. et al. 2003. Implementation of Noah land surface model advances in the National Centers for Environmental Prediction operational mesoscale Eta model. *Journal of Geophysical Research: Atmospheres.* **108**(D22).

Emmons, L.K. et al. 2010. Description and evaluation of the Model for Ozone and Related chemical Tracers, version 4 (MOZART-4).

Gettelman, A. et al. 2019. The whole atmosphere community climate model version 6 (WACCM6). *Journal of Geophysical Research: Atmospheres.* **124**(23), pp.12380-12403.

Grell, G.A. and Dévényi, D. 2002. A generalized approach to parameterizing convection combining ensemble and data assimilation techniques. *Geophysical Research Letters.* **29**(14), pp.38-1-38-4.

Hersbach, H. et al. 2020. The ERA5 global reanalysis. *Quarterly Journal of the Royal Meteorological Society.* **146**(730), pp.1999-2049.

Iacono, M.J. et al. 2008. Radiative forcing by long-lived greenhouse gases: Calculations with the AER radiative transfer models. *Journal of Geophysical Research: Atmospheres.* **113**(D13).

Morrison, H. et al. 2009. Impact of cloud microphysics on the development of trailing stratiform precipitation in a simulated squall line: Comparison of one-and two-moment schemes. *Monthly weather review.* **137**(3), pp.991-1007.

Nakanishi, M. and Niino, H. 2006. An improved Mellor–Yamada level-3 model: Its numerical stability and application to a regional prediction of advection fog. *Boundary-Layer Meteorology.* **119**(2), pp.397-407.

Tie, X. et al. 2003. Effect of clouds on photolysis and oxidants in the troposphere. *Journal of Geophysical Research: Atmospheres.* **108**(D20).
